# Advanced Research on Immune Checkpoint Inhibitor Therapy

**DOI:** 10.3390/jcm11185392

**Published:** 2022-09-14

**Authors:** Hisao Imai, Kyoichi Kaira, Hiroshi Kagamu

**Affiliations:** Department of Respiratory Medicine, International Medical Center, Saitama Medical University, Hidaka 350-1298, Saitama, Japan

The human body has an inherent immune surveillance mechanism that eliminates cancer cells and suppresses the development of cancer. In 2002, Dunn *et al.* proposed “cancer immunoediting” [1]. Cancer immunoediting consists of three phases: an “elimination phase” in which cancer cells are completely eradicated; an “equilibrium phase” in which cell proliferation is suppressed, controlled by a small number of cells for years; and an “escape phase” in which cell proliferation is allowed and the cell number reaches clinically detectable levels. Although immune surveillance mechanisms against cancer cells are composed of various immune cells, it was elucidated that the equilibrium phase is maintained by T-cell immunity, which consists of CD8^+^ T cells that recognize MHC class Ⅰ-restricted antigens expressed on cancer cells and directly kills them and CD4^+^ T cells that assist in priming, clonal expansion, migration, tumor infiltration, cell-killing activity, and the survival of CD8^+^ T cells. Cancer immune escape mechanisms include the loss of tumor antigen/HLA expression, the expression of immunosuppressive substances, the induction of immunosuppressive cells, and the T-cell exhaustion phenomenon [2]. Programmed cell death protein 1 (PD-1), discovered by Honjo *et al.* in 1992, was found to be a regulator of T cell signaling, and plays a central role in the T-cell exhaustion phenomenon [3,4]. Subsequently, a PD-1 inhibitory antibody that blocks PD-1 signaling was used in a mouse model to confirm its effect on cancer shrinkage, proving for the first time that the interaction between PD-1 and its ligand, the programmed cell death ligand 1 (PD-L1), plays an important role in the immune escape mechanism in cancer [5]. Immune checkpoint inhibitors (ICIs), including antibodies to the cytotoxic T lymphocyte antigen 4 (CTLA-4), PD-1, and PD-L1, promote the antitumor function of T cells [6].

To date, one anti-CTLA-4 antibody (ipilimumab), three anti-PD-1 antibodies (pembrolizumab, nivolumab, and cemiplimab), and three anti-PD-L1 antibodies (atezolizumab, avelumab, and durvalumab) have been approved for the treatment of different malignancies by the United States Food and Drug Administration (FDA) [7]. Ipilimumab, a monoclonal antibody against CTLA-4, was approved by the FDA in 2011 for patients with metastatic melanoma [8]. It was the first clinically approved immune checkpoint inhibitor. Since then, cancer immunotherapies, including PD-1/L1 and CTLA-4 antibodies, have revolutionized cancer treatment. ICIs have produced favorable results in various clinical trials and are currently being used in clinical practices for the treatment of malignant melanoma, lung cancer, renal cell carcinoma, Hodgkin’s lymphoma, head and neck cancer, gastric cancer, urothelial carcinoma, and other cancers [6].

In clinical practice, ICIs show characteristic anti-tumor effects in multiple cancers and promote long-term survival in some patients, which is difficult to achieve with conventional therapy; however, some patients hardly experience any benefit. The details of the mechanism responsible for such significant differences in therapeutic efficacy are not yet clear. Extensive trials have been conducted over the past decade on the role of ICIs and are paving the way for an era of immunotherapy in cancer treatment [9,10]. In the future, optimal tumor-specific combination therapies are likely to be selected, further improving their therapeutic effect. However, there are currently several important problems with cancer immunotherapy. The efficacy of ICIs is quite limited among biliary cancer, pancreatic cancer, and prostate cancer [11,12,13]. Furthermore, although several biomarkers—such as PD-L1 expression, tumor mutational burden (TMB), microsatellite instability (MSI), and tumor-infiltrating lymphocytes (TILs)—that may be useful as therapeutic predictors are used in practice, the lack of established predictive biomarkers that can reliably select treatment choices is an important clinical question regarding ICIs [14,15,16]. Immune-related adverse events (irAEs), which are unique to ICIs, involve an excessive immune response to the self-antigens induced by the administration of ICIs and are an important clinical issue because they can occur at various times and in different forms, sometimes resulting in fatal adverse events [17]. In such cases, important clinical problems, such as the lack of biomarkers to predict the therapeutic efficacy of ICIs, whether ICI monotherapy or combination therapy is more effective, and the influence of steroids on the efficacy of ICIs, must be clarified in clinical settings using ICIs.

This Special Issue in the Journal of Clinical Medicine focuses on “Advanced Research on Immune Checkpoint Inhibitor Therapy” and aims to identify important open questions and future perspectives in contemporary cancer immunotherapy, including ICIs and combination therapies, potential biomarkers to predict treatment efficacy, real-world clinical efficacy and safety considerations, and other timely and emerging topics. International experts in the field have examined key approaches actively being investigated in clinical and preclinical research and have integrated their individual perspectives into their publications. This Special Issue presents a series of three papers (an original article and two reviews) by international leaders in the field.

Ushio and colleagues have reviewed predictive ICI biomarkers for non-small cell lung cancer (NSCLC) [18]. Currently, the reality is that some NSCLC patients do not respond to ICIs and predictive biomarkers other than tumor PD-L1 expression have not been established. Therefore, ICI biomarker identification is an urgent issue. The review article outlines the current understanding of predictive markers for the efficacy of ICIs, including PD-L1, TMB, DNA mismatch repair deficiency, MSI, CD8^+^ TILs, human leukocyte antigen class I, tumor/specific genotype, and blood biomarkers, such as the peripheral T-cell phenotype, the neutrophil-to-lymphocyte ratio, interferon-gamma, and interleukin-8. Kim and colleagues reviewed a Bayesian network meta-analysis to suggest front-line treatment for advanced NSCLC showing high PD-L1 expression [19]. The analysis of 5237 patients from 22 studies shows that ICI monotherapy may be reasonable as a first-line treatment for advanced NSCLC with high PD-L1 expression. Thanks to Mouri and colleagues, new findings on the safety of ICI treatments were also obtained [20]. Their original article reports the effect of systemic steroid use on irAEs in NSCLC patients being treated with PD-1 inhibitors. The study shows that patients who received systemic steroids for irAEs owing to PD-1 inhibitor therapy had shorter progression-free survivals compared to those who did not receive systemic steroids. This suggests that systemic steroids may affect survival after a PD-1 blockade, even for patients who once acquired a clinical benefit from them.

In terms of optimal patient selection and prognostication, special attention should be paid to treatment choices, such as whether ICI monotherapy or combination therapy is the best therapeutic approach, and the impact of steroids used for irAE on the therapeutic efficacy of ICIs. Regarding the biomarkers for ICIs, while considerable progress has been made, much remains to be accomplished to achieve an even greater accuracy. While it is clear that the debate over the best therapies, biomarkers, and irAEs in the era of ICIs is far from over, this Special Issue will help shed light on the debate regarding ICI therapy.
